# Carbamazepine induced toxic epidermal necrolysis and Stevens-Johnson syndrome overlapping during pregnancy in a South-East Asian patient: A case report

**DOI:** 10.1016/j.amsu.2021.102616

**Published:** 2021-07-27

**Authors:** Oshan Shrestha, Prashant Pant, Nebula Devkota, Dhiraj Gurung, Dhan Bahadur Shrestha

**Affiliations:** a Nepalese Army Institute of Health Sciences, Kathmandu, Nepal; b Department of Emergency Medicine and Critical Care Unit, Karnali Academy of Health Sciences, Jumla, Nepal; c Department of Internal Medicine, Karnali Academy of Health Sciences, Jumla, Nepal; d Department of Emergency Medicine, Mangalbare Hospital, Morang, Nepal

**Keywords:** Carbamazepine, Toxic epidermal necrolysis, Steven- Johnson syndrome, South-East Asia

## Abstract

**Introduction:**

Toxic Epidermal Necrolysis (TEN) and Stevens-Johnson syndrome (SJS) are rare and severe forms of drug-induced skin reaction. Most frequently involved drugs are noted to be non-steroidal anti-inflammatory agents, antibiotics, and anticonvulsants. These have high morbidity and mortality and counts among dermatological emergencies.

**Case presentation:**

We report an eventful case of a 22-year-old lady who suffered and recovered from carbamazepine-induced SJS/TEN overlapping during her pregnancy. Our patient had a history of epilepsy for which she was under sodium valproate. Switching to carbamazepine due to its low teratogenicity led our patient to this condition. History of prodromal symptoms and exposure to carbamazepine helped in the diagnosis. Carbamazepine abstinence and a multidisciplinary approach in symptomatic management worked very well for the patient.

**Clinical discussion:**

Carbamazepine-induced TES/SJS manifests multisystem effects and requires a multidisciplinary approach for management. The condition itself is life-threatening and in its addition, their sequelae further threaten the life of the patients. Early intervention is the key. Genetically susceptible are thought to be the ones carrying human leukocyte antigen B*15:02 (HLA-B*15:02) allele and it is most prevalent in South-East Asian populations. Screening of this allele before using carbamazepine prevents the incidence of carbamazepine-induced SJS/TEN.

**Conclusion:**

Prodromal symptoms of carbamazepine-induced SJS/TEN constitute flu-like symptoms that should not be missed. Early intervention and multidisciplinary approach prevent secondary infections and complications. Screening for HLA-B*15:02 variant allele and close monitoring of these adverse reactions along with proper counseling to patients goes a long way in preventing the development of this life-threatening condition.

## Highlights

• Carbamazepine-induced cutaneous reaction may present as Stevens-Johnson Syndrome (SJS) or toxic epidermal necrolysis (TEN).
• Carriers of the human leukocyte antigen B*1502 gene are susceptible to developing carbamazepine-induced SJS/TEN.
• Human leukocyte antigen B*1502 gene is most prevalent in South-East Asia.
• SJS/TEN affect multisystem and need a multidisciplinary approach in management.

## 1 Introduction

Alan Lyell, in the year 1956, described four cases of skin eruptions. Out of those four reported cases, one that had a history of drug ingestion is called Toxic Epidermal Necrolysis (TEN) or Lyell's syndrome in today's world [1,2]. TEN is the severe and rare form of drug-induced skin reaction and counts among dermatological emergencies. The onset of these conditions is attributed to preceding medications like non-steroidal anti-inflammatory agents, antibiotics, and anticonvulsants most frequently [2]. Such adverse drug reactions may occur as Stevens-Johnson syndrome (SJS) or Toxic Epidermal Necrolysis (TEN). SJS/TEN are seen as two ends of a spectrum of a severe and life-threatening form of drug-induced skin reaction, differing only in body surface area (BSA) involvement (SJS: <10 %, SJS/TEN overlap: 10–30 %, TEN: >30 %) [3].

Carbamazepine was found to be the commonest cause of SJS/TEN in a retrospective study [4]. According to carbamazepine adverse drug reaction (ADR) classification of the World Health Organization (WHO), SJS/TEN falls under type B adverse effect having genetic and immunological predisposition. SJS/TEN are the type of ADR that are rare, unpredictable, and presents with high morbidity and mortality [5].

Here we report an eventful case of a 22-year-old pregnant female who suffered then recovered from carbamazepine induced SJS/TEN and had a spontaneous abortion during its peak before recovery. This case report is in line with the SCARE 2020 criteria [6].

## 2 Case presentation

A 22 yr old multigravida (G3P2L1A1) from Jumla, Nepal was brought to the emergency department of Karnali Academy of Health Sciences (KAHS) and admitted with the concerns of multiple erythematous papular rashes with fluid-filled vesicles that had erupted over her face, then on her neck, arms, thorax and back regions. The patient was the first in her family to develop such eruptions and she has no known history of allergies.

Thorough history revealed that the patient is a diagnosed case of epilepsy for which she has been taking sodium valproate since she was 13 years of age. Eighteen days before the admission, the patient had gone for her antenatal care (ANC) visit where she was switched to carbamazepine due to its low teratogenicity compared to sodium valproate. On the 8th day of use of carbamazepine, she developed a cough, sore throat, malaise, fever, and loss of appetite followed by irritation over the eyes. Then, on the 12th day of exposure, in addition to all the above symptoms, she developed erythematous rashes and fluid-filled vesicles that firstly appeared on her face, then spread to the neck, arms, thorax, and back regions.

The patient was brought to the hospital on the 19th day of exposure, before that she visited faith healers. At this time we noticed that she had multiple erythematous papular lesions with fluid-filled vesicles/bullae over her face, then on her neck, arms, thorax, and back regions. She had areas of denuded skin with BSA involvement of 21 % (face: 9 %, right and left upper arm: 2 % + 2 %, back: 2 %, chest and abdomen: 2 % + 2 %, right and left lower limb: 2 %). Nikolsky's sign was positive. Also, ocular examination revealed matted cilia with blepharitis and congested conjunctiva with normal findings in the fundus. Whereas oral mucosa and vermillion border showed painful hemorrhagic erosions, and also, the tongue was edematous. The patient was in pain and couldn't swallow for which a nasogastric tube was introduced.

On the 2nd day of admission, in her 8 weeks and 3 days of gestation, she had per-vaginal bleeding and fleshy clot were passed which was suggestive of spontaneous abortion. So, suction and evacuation were carried out (Photograph 1, Photograph 2, Photograph 3, Photograph 4).

**Photograph 1:**
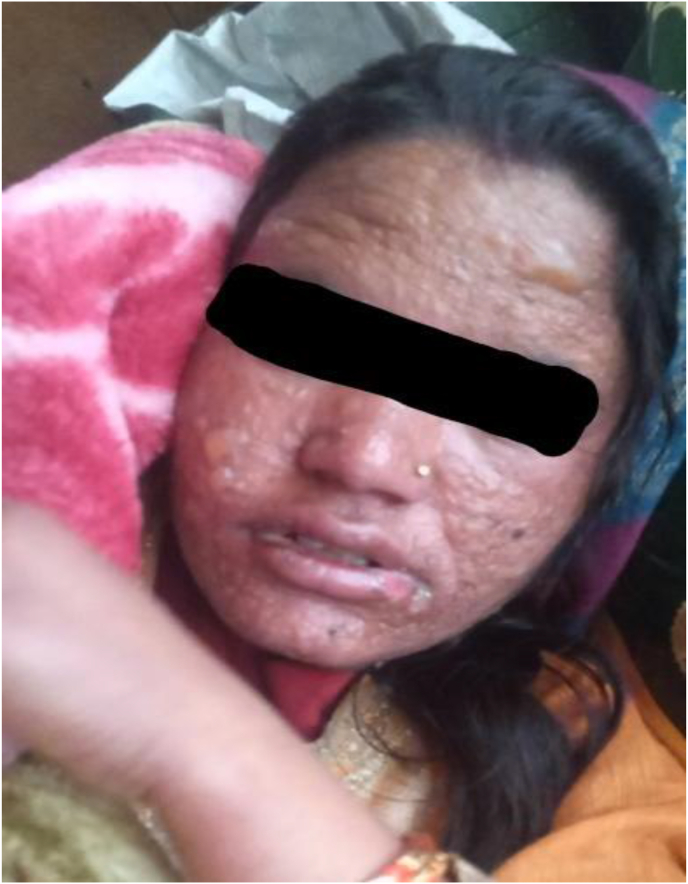
Rash and vesicles over face on the 12th day of exposure to carbamazepine.

**Photograph 2:**
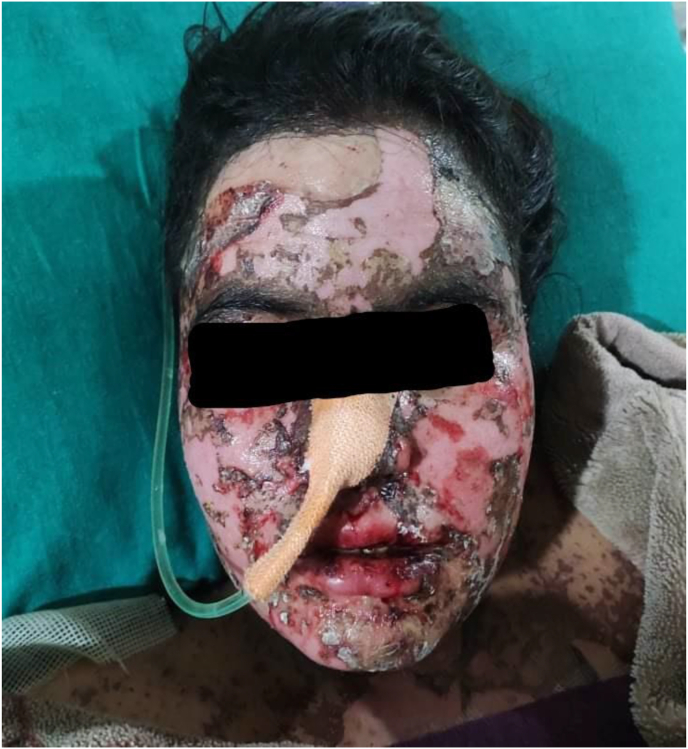
Areas of denuded skin and hemorrhagic erosions over face on the 19th day of exposure.

**Photograph 3:**
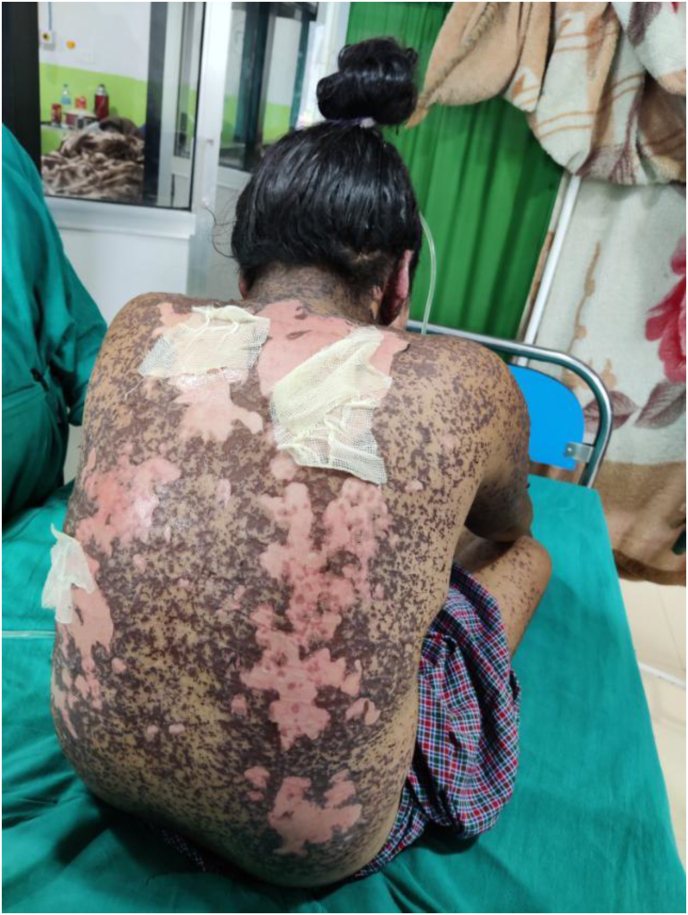
Areas of denuded skin over back on 19th day of exposure.

**Photograph 4:**
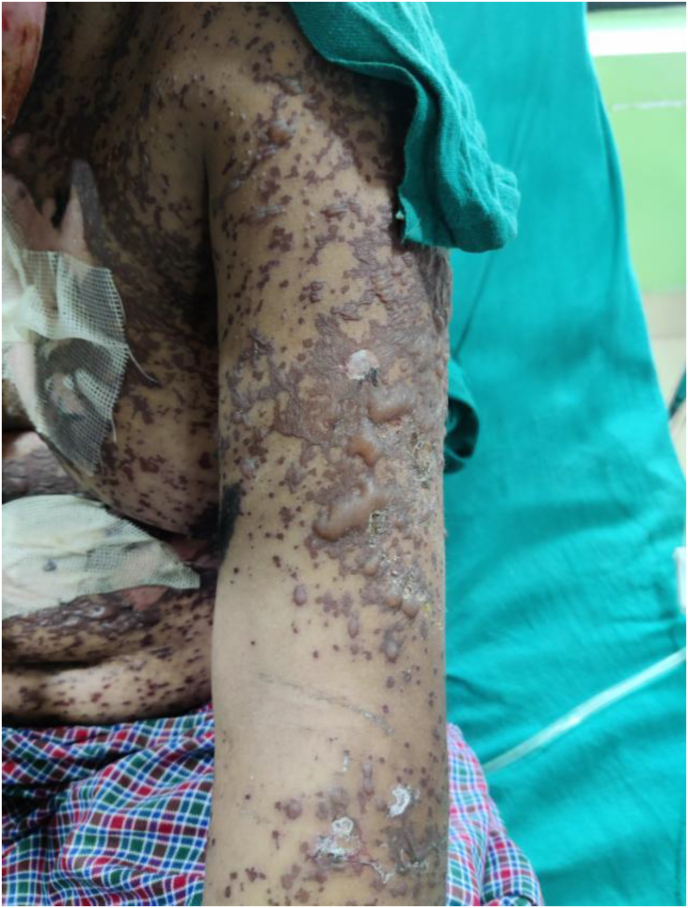
Rash and vesicles/bullae over left arm on the 19th day of exposure.

### 2.1 Timeline

**Fig. 1:**
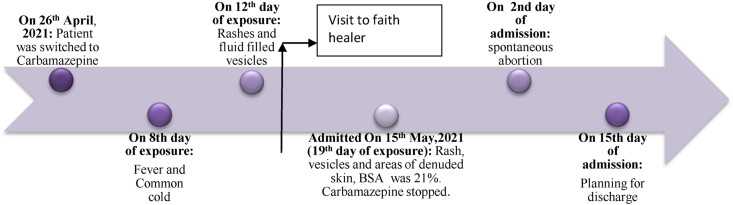
Timeline of events.

First sign of ADR was seen on the 8th day of exposure and the worst with 21 % BSA involvement was seen on the 19th of exposure to carbamazepine (Fig. 1)

### 2.2 Diagnostic assessment

At the time of presentation, body temperature was 37.33° Celsius, blood pressure was 100/70 mm Hg, pulse rate was 128/min and respiratory rate was 22/min. Test of C-reactive protein came positive and erythrocyte sedimentation rate (ESR) was 50mm/hr. Aspartate aminotransferase (AST) and alanine aminotransferase (ALT) measured 44.5 u/l and 50.3 u/l respectively. Other parameters were not deranged and serological tests to rule out infections (human immunodeficiency virus, hepatitis B virus, and *Treponema pallidum*) came out negative. Considering the history of exposure to carbamazepine, prodromal symptoms, nature of the lesions, and laboratory reports, diagnosis of SJS/TEN overlapping was made.

### 2.3 Treatment

Carbamazepine was stopped immediately and sodium valproate (200mg, per-oral, once a day) was reinstated. The patient was put on chlorpheniramine (20mg for 7 days), ceftriaxone (1 gm for 14 days), dexamethasone (6mg for 10 days), metronidazole (500mg for 10 days), and paracetamol (500mg for 14 days), all intravenously once in a day. Intravenous fluid was given by maintaining a strict in/out chart. Eye care was done for fourteen days through the administration of eye drops of carboxymethylcellulose (0.5 %) and eye drops having a combination of chloramphenicol (4 mg/ml), polymyxin B (5000 IU/ml), and dexamethasone (1 mg/ml). For oral care chlorhexidine (0.2 %) gargle and topical gel having a combination of lidocaine (2 %), chlorhexidine gluconate (1 %), and metronidazole (1 %) were used. Mupirocin (2 %) ointment was applied in skin lesions after dressing for two weeks. As prophylaxis enoxaparin (40mg, subcutaneously, for 10 days) and ranitidine (30mg, intravenously, in two divided doses for 2 weeks) were used. Abstinence to carbamazepine and supportive measures have worked well for the patient. The patient is recovering well with no trace of new issues and adverse reactions (Photograph 5).

**Photograph 5:**
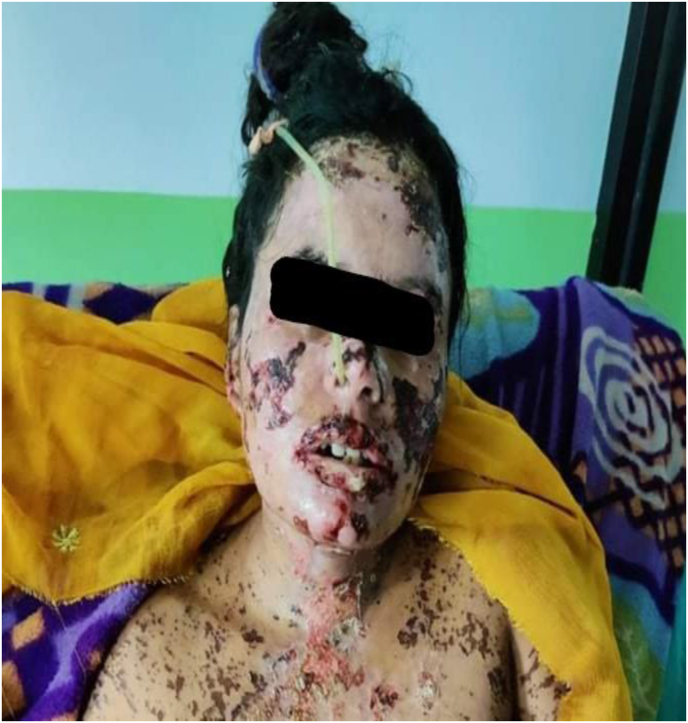
Healing of cutaneous and oral mucosal lesions on the 15th day of abstinence to carbamazepine.

### 2.4 Follow-up

The patient and her family were well counseled about her clinical state and the medications she was put under. Discharge is being planned for the patient with sodium valproate tablets (200mg), chlorhexidine(0.2 %) gargle, mupirocin(2 %) ointment and pantoprazole tablets (40mg for 10 days). She will be asked to visit for a follow-up after two weeks for assessments.

## 3 Discussion

The role of carbamazepine in inducing TES/SJS is well known. It onsets after 4–21 days of exposure and affects those with immunological and genetic susceptibility [5]. This may be a rare adverse effect but it is well documented and advocated at today's date. Our patient was switched to carbamazepine from sodium valproate as it is a safer choice during pregnancy having lower teratogenic risk [7] but the fact that the patient went to faith healers after the onset of prodromal symptoms reflects that she was not counseled about the adverse effects of carbamazepine. Early presentation to the hospital saves the patient from physical and mental havoc which is achieved through proper counseling.

TEN/SJS doesn't only raise dermatological concerns. Yip et al. have reported that 50 % of patients experience late ocular complications, in descending order of frequency, dry eyes, trichiasis, symblepharon, distichiasis, visual loss, entropion, ankyloblepharon, lagophthalmos, and corneal ulceration [8]. Our patient had blepharitis and congested conjunctiva on the day of presentation. Ocular care with ophthalmic lubricant, antibiotics, and corticosteroid helped to stop further progression. The patient was also put under enoxaparin as prophylaxis for thrombus formation. Siddika et al. reported a rare case of reactive thrombocytosis that occurred in SJS leading to hyperviscosity syndrome [9]. SJS and TEN, affecting multisystem and requiring multidisciplinary approach, are life-threatening and in its addition, their sequelae further threaten the life of patients. Early intervention is the key and proper counseling to the patients always goes a long way in early diagnosis and avoiding life-threatening circumstances.

Genetically susceptible are thought to be the ones carrying human leukocyte antigen B*15:02 (HLA-B*15:02) allele, a variant of HLA-B. Its relationship with carbamazepine-induced SJS/TEN is well established and it is most prevalent in Asian populations (Indians, Han Chinese, Thais, and Malays) [10,11]. There is no such data available of Nepal but considering the vicinity, there is a high possibility of this variant allele being prevalent in Nepal. Many studies have concluded that screening of HLA-B*15:02 allele before using carbamazepine prevents the incidence of carbamazepine-induced SJS/TEN [[10], [11], [12]].

To put light on the spontaneous abortion that our patient had to bear, we ruled out other causes but to attribute this to carbamazepine or SJS/TEN we couldn't find any concrete evidence. There is a need for studies to fill the gap between spontaneous abortion and carbamazepine-induced ADR to distinguish this event as a clinically significant event or a mere coincidence.

## 4 Conclusion

Flu-like symptoms post carbamazepine exposure should not be overlooked as they can be prodromal symptoms of SJS/TEN. A multi-disciplinary approach and early intervention prevent secondary infections and complications. Screening for HLA-B*15:02 variant allele or if not available, close monitoring of these adverse reactions and proper counseling to patients is very necessary considering high mortality and morbidity of SJS/TEN. Pharmacovigilance for the identification, assessment, and prevention of ADRs can provide continuous information on the safety and appropriate use of the drug.

## Patient perspective

Our patient grew positively when her patience and our treatment started yielding good results. The patient's insight and thoughts regarding her condition have changed and she is now aware that the difficult condition she had to bear was due to exposure to carbamazepine. Our patient regrets not seeking medical attention at the initial stage and is thankful to the whole medical fraternity for helping her to recover.

## Consent of the patient

Written informed consent was obtained from the patient for publication of this case report and accompanying images. A copy of the written consent is available for review by the Editor-in-Chief of this journal on request.

## Author agreement statement

This is an original work that was completed by our respective substantial contributions. We solemnly declare that this manuscript has not been published before nor this is in consideration for publication in other journals. We also confirm that all the mentioned authors are aware of all the declarations and agree to them.

## Provenance and peer review

Not commissioned, externally peer-reviewed.

## Declaration of competing interest

No conflicts of interest.

## Registration of research studies

1. Name of the registry: N/A.
2. Unique Identifying number or registration ID: N/A.
3. Hyperlink to your specific registration (must be publicly accessible and will be checked): N/A.
